# Nutrition Interventions of Herbal Compounds on Cellular Senescence

**DOI:** 10.1155/2022/1059257

**Published:** 2022-04-27

**Authors:** Zhongxu Chen, Yixin Wu, Qinlu Lin, Jie Cai, Xi Liu, Ying Liang

**Affiliations:** Molecular Nutrition Branch, National Engineering Research Center of Rice and By-product Deep Processing, College of Food Science and Engineering, Central South University of Forestry and Technology, Changsha, 410004 Hunan, China

## Abstract

When cells undergo large-scale senescence, organ aging ensues, resulting in irreversible organ pathology and organismal aging. The study of senescence in cells provides an important avenue to understand the factors that influence aging and can be used as one of the useful tools for examining age-related human diseases. At present, many herbal compounds have shown effects on delaying cell senescence. This review summarizes the main characteristics and mechanisms of cell senescence, age-related diseases, and the recent progress on the natural products targeting cellular senescence, with the aim of providing insights to aid the clinical management of age-related diseases.

## 1. Introduction

Aging is not regarded as a disease but rather as a unique and independent pathological state. It precedes the onset of many other diseases and is an inevitable biological process. Aging is a multifactor universal process that occurs at the molecular, cellular, and tissue levels. It is characterized by the loss and degeneration of constituent materials, tissue structures, and physiological functions in the body [1, 2].

Research has demonstrated the important role of cellular senescence in the aging process [3, 4]. Cellular senescence was first described as permanent cell cycle arrest when cells reach their replication limit (replication senescence). Even under suitable growth conditions, senescent cells no longer divide and the cell cycle enters an irreversible arrested state [5]. During aging, persistent DNA damage response (DDR) markers and senescence-associated secretory phenotype (SASP) are accumulated in terminally differentiated cells [6]. Cellular senescence also plays a physiological role in the normal development of the body, such as in combination with apoptosis to promote embryonic morphological development [7, 8]. In mature tissues, cellular senescence is mainly triggered by response to injury, thereby inhibiting potentially dysfunctional cells. However, over time, the abnormal accumulation of senescent cells can cause harmful effects [9]. Cellular senescence is the main mechanism that may lead to chronic diseases and age-related dysfunction [10]. In vitro experiments in cells are an important method to study cellular senescence, and these cell experiments may help provide insights into the relationship between senescence and age-related human diseases.

## 2. Main Indicators of Cell Senescence and the Potential Mechanism

Currently, no universal marker is available to detect cell senescence [11]. Given that biological markers expressed by senescent cells might vary with cell type, stimulation, and stimulation duration, several senescence-related markers need to be evaluated to consolidate the cell senescence phenotype [12].

### 2.1. Morphological and Metabolic Changes in Senescent Cells

**Figure 1:**
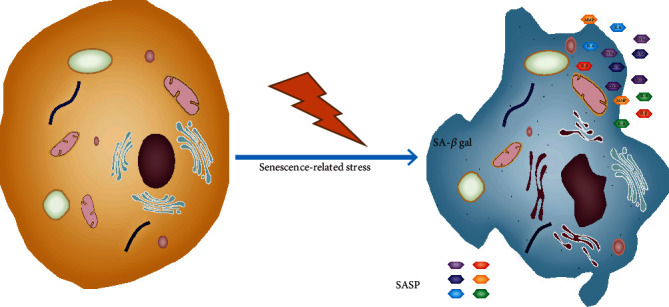
Morphological and metabolic changes in senescent cells. Markers of cell senescence include changes in *β*-gal activity caused by increased lysosome content and activity, the loss of lamin B1 caused by the changes in the nuclear envelope, the increase of lipofuscin labeled by Sudan black B staining, and morphological changes, such as flat cell bodies. Senescence-associated secretory phenotype- (SASP-) related factors, such as TNF*α*, IL-1*α*, IL-1*β*, and matrix metalloproteinase (MMP) and loss of nuclear localization of HMGB1 are also common markers of senescence.

The morphology of senescent cells is drastically different compared with that of normal cells (Figure 1). During senescence, cell density decreases and cells undergo morphological changes [ [13]]. Compared with normal cells, senescent cells typically display an enlarged although flattened shape. The intercellular boundaries of senescent cells become inconspicuous and extensive vacuolization occurs. The integrity of nuclear membranes is damaged due to the loss of lamin B1 expression. The nuclear membrane collapses and chromatin agglutination and pyknosis occur [14]. Senescent cells accumulate defective mitochondria and increased levels of reactive oxygen species (ROS). In senescent human cells, the content of lysosomes increases and lysosomal activity changes, which is manifested by an increase in *β*-galactosidase (*β*-gal) activity at pH 6 [15]. This specific *β*-gal activity was the first and is one of the most widely used gold standards for evaluating cellular senescence [16].

As the first evidence for *β*-gal accumulation in cell senescence, Dimri et al. noted increased levels of *β*-gal in epidermal cells from the skin with age [15]. *β*-Gal is also expressed in certain nonsenescent cells, including osteoclasts and mature macrophages, under normal physiological conditions [17]. Changes in conditions, such as pH and incubation duration, can stimulate some normal cells to exhibit false-positive results [18]. *β*-Gal, which is rarely seen in a neutral pH environment under normal conditions, shows high enzymatic activity within 1 h in response to ionizing radiation [19]. Recently, Cai and colleagues identified a new prodrug SSK1 that is specifically cleaved by lysosomal *β*-gal into cytotoxic substances to stimulate apoptosis and the elimination of senescent cells. In aged mice, SSK1 eliminated senescent cells in various tissues, reduced levels of senescence-related genes such as p16 and p21, reduced mild local and systemic inflammation, and restored organismal function [20]. These findings indicate that lysosomal *β*-gal may represent an effective target for the selective elimination of senescent cells, providing a new strategy for the development of antisenescence drugs.

Recently, metabolomics analysis on human umbilical vein endothelial cells (HUVECs) was carried out, from the third to eighteenth population doublings, and enriched 14 overtly changed metabolic pathways in senescent cells [21]. This work provided a new perspective to understand the mechanism of cell senescence.

### 2.2. p16^Ink4a^

**Figure 2:**
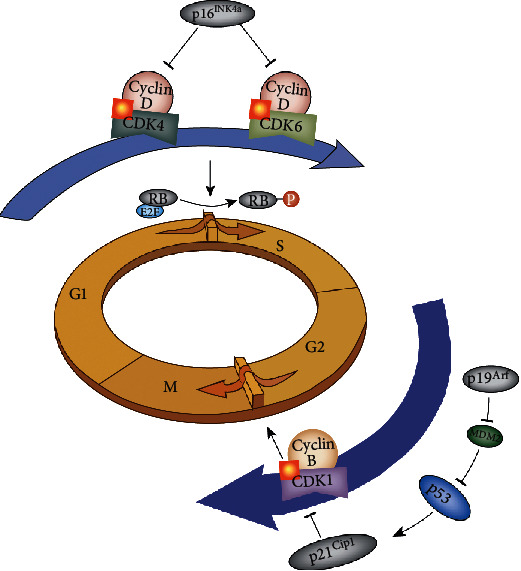
The main regulators of the cell cycle and their functions in senescence. The cyclin-dependent kinase inhibitors p16^INK4A^ and p21^Cip1^ are commonly used markers of senescence. Cell cycle arrest is induced by the inhibition of cyclin-dependent kinases (CDKs) through the p53/p21^Cip1^ and/or Rb/p16^INK4A^ pathways, which causes sustained DNA damage.

p16^Ink4a^ is a cyclin-dependent kinase inhibitor that competitively binds with CDK4/6, thus inhibiting phosphorylation of the main substrate Retinoblastoma (Rb) [22]. Rb in the nonphosphorylated state binds to the transcription factor E2F, thus inhibiting the expression of genes. The expression of p16^Ink4a^ increases with an increased number of cell divisions [23]. In response to stress factors, most cells trigger senescence through the p16^Ink4a^-Rb signaling cascade [24]. This process arrests the cell cycle in the G1/S phase, which leads to cell senescence [25] (Figure 2). Notably, approximately 75% of human cancer cell lines contain mutations or deletions in the p16 gene, which prevents these cells from entering the senescence process. Therefore, p16 expression is used as a cell senescence biomarker.

A recent study used p16^tdTom^ as a reporter allele and sensitive tool to count, isolate, and identify single cells that expressed p16^INK4a^ [26]. Grosse and colleagues developed a knock-in strategy to target p16 and monitor senescence. The authors found that p16^Ink4a^ was rarely expressed in healthy, stress-free tissues and cells in young animals. As mice age (10–12 months old), an increasing number of cells in tissues undergoing senescence, damage, and initial tumorigenesis start to express p16^Ink4a^ [27]. Elevated expression of p16 may be a marker to eliminate senescent cells in mice with a prolonged lifespan. However, this type of method does not appear to be particularly accurate since p16 cells are not eliminated in the colon, liver, and lymphocytes [28]. Childs and colleagues described endothelial cells from the p16-positive p16-3MR transgenic mice as senescent cells. Following ganciclovir treatment in these mice, atherosclerotic plaques were retarded [29]. While there is currently no method for targeting senescent cells that is very precise, targeting p16 may be one of the best methods.

### 2.3. p21^Cip1^

The cyclin-dependent kinase inhibitor p21^Cip1^, which is a transcriptional target of the p53 tumor suppressor, regulates the cell cycle by binding and inhibiting its partner cyclin, leading to the inhibited transition of cells from G1 to S phase and from G2 to M phase [30–32] (Figure 2). The p53 tumor suppressor is inactivated in most tumors, and its expression is upregulated in senescent cells. p53 protein levels are mostly regulated by ubiquitin-mediated proteosomal degradation [33]. The MDM2 ubiquitin ligase, which is highly expressed in most tumors, directly binds with p53 protein to suppress p53 transcriptional activity and promotes the degradation of p53 by ubiquitin-mediated degradation [34]. The p19^Arf^ protein, which is encoded by the Arf gene locus that overlaps with the Ink4a gene locus, binds and inhibits MDM2 activity, subsequently activating p53 signaling [35]. Upon DNA damage (e.g., ionizing radiation and telomere dysfunction), p19^Arf^ is upregulated to inhibit MDM2 and activate p53, which results in the induction of the p53 downstream target p21^Cip1^. p21^Cip1^ also functions in the inhibition of Rb phosphorylation; as described above, once phosphorylated, Rb cannot bind with E2F, which leads to cell cycle arrest in the G1 phase and cell senescence [36]. Chakraborty et al. reported a senescence characteristic cell phenotype in pancreatic and breast cancer cells treated with erythronol. The authors found that *β*-gal activity increased along with elevated expression of p21 and decreased amounts of CDK2 and cyclin D1 [37].

### 2.4. Telomere Shortening

Research has shown that telomeres are damaged and become shortened as cells divide [38], and the shortening or destruction of telomeres plays a critical function in influencing cell senescence [39]. Telomerase is a reverse transcriptase that is mainly responsible for telomere lengthening, completely independent of replication [40]. Telomerase activity is inhibited or lost after oxidative damage [41–44], leading to the loss of the telomeric ends in the chromosome replication process, therefore accelerating cell senescence [45]. Fouquerel et al. used a targeted combination of telomeres and photochemically generated singlet oxygen to selectively control the time and length of oxidative stress applied to telomere sites [38]. The authors repeatedly exposed cultured cancer cells to this targeted oxidation process to simulate environmental stress and inflammatory conditions. In fact, even though telomerase, which is responsible for telomere lengthening, was reactivated, the telomeres still shorten as the cells divided. Quratul and colleagues found that in mouse cortical nerve cells, the primary reason for telomere shortening with age is not due to telomerase activity (which remains almost constant) but may be from changes in the telomere reverse transcriptase protein content (rather than the RNA component) in mouse cortical nerve cell subchambers. The hTERT component of telomerase selectively increases in cytoplasmic and membrane-bound portions with age [46]. Galbiati et al. used DNA in situ bridging to detect DNA breakage sites and analyzed adjacent sites [47]. This new localization method might detect extreme telomeres that are present in cells. However, in some nonsenescent cells expressing p16^Ink4a^, telomere shortening and the loss of telomere function could also be detected, and some stress-induced cellular senescence was independent of the telomere shortening pathway [48–50]. Although telomere shortening is detrimental for healthy cells, targeting telomeres in tumor cells represents a method to fight cancer. Drugs that activate and regulate telomerase have been developed with the aim of designing intervention strategies to protect telomeres in healthy cells and target telomeres in cancer cells.

### 2.5. Senescence-Associated Secretory Phenotype- (SASP-) Related Factors

During senescence, cells secrete many active substances, such as soluble signaling messengers, proteases, and extracellular matrix proteins. Among them, soluble factors, such as cellular inflammatory factors, chemotactic cytokines, growth factors, and immunoregulatory factors, promote cell proliferation and inflammatory responses by changing the microenvironment surrounding cells and promoting the cancerous transformation of cells [51]. For example, IL-6, one of the important SASP factors that is directly regulated by DNA damage signaling, is closely associated with cellular senescence [52, 53]. SASP factors exhibit a dual regulatory role. SASP factors induce activation of the immune system to clear senescent cells and the growth stagnation of senescent cells and participate in tumor suppression. In addition, SASP factors secreted by senescent cells are involved in the destruction of normal tissue structures, induce epithelial-mesenchymal transition, and promote the proliferation of malignant tumors [54]. In some cases, however, this property of senescent cells may help protect the body in specific conditions. For example, following hemorrhagic shock in rats, liver cells immediately enter a state of senescence to prevent organ failure, preserving organismal homeostasis [55]. This could explain the distinct selection mechanisms by which immune cells eliminate senescent cells; senescent cells that promote the secretion of inflammatory substances are eliminated and cells induced to undergo senescence for protective mechanisms may not be eliminated. However, further research is required to address this possibility.

## 3. Diseases Related to Cellular Senescence

**Figure 3:**
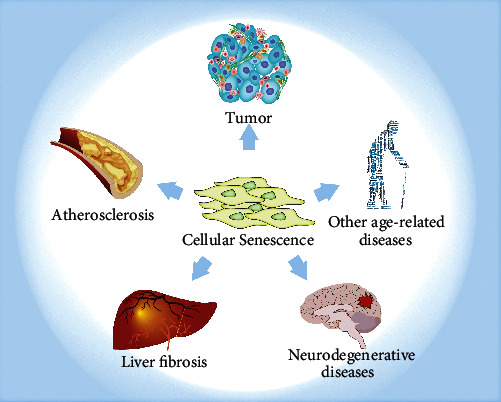
Diseases related to cellular senescence. Although cellular senescence is a normal process during development and tissue remodeling, it is related to a decline in tissue function and various disease states. These diseases include but are not limited to cancer, atherosclerosis, liver fibrosis, neurodegenerative diseases, and other diseases.

Senescent cells lose their ability to divide and undergo apoptosis and remain in the body [56]. Accumulation of senescent cells is associated with a series of age-related diseases [22], such as cancer, atherosclerosis, liver fibrosis, and neurodegeneration [57] (Figure 3). Therefore, better understanding of how senescent cells affect these diseases and the development of methods to eliminate accumulated senescent cells could be of significant potential for the management of many age-related pathologies.

### 3.1. Cancer

The relationship of cellular senescence with cancer varies depending on the physiological environment [58]. Cellular senescence in the early stage of tumorigenesis can reduce the incidence of cancer. Cellular senescence involves an irreversible block of cell proliferation, which also represents a powerful mechanism for autonomously inhibiting cancer [59]. At the late tumor stage, senescent cells eventually show complex, multicomponent SASP. SASP alters the behavior of adjacent cells and the tissue microenvironment. A notable feature of SASP is the large number of proinflammatory factors, including chemokines, cytokines, and damage-associated molecular patterns (DAMPs). Chronic inflammation, as a common feature of aging tissues, is a major risk factor for cancer in later life [5, 60].

Oncogenes induce cellular senescence in the early stage of tumorigenesis. Senescent cells secrete active substances that alter the microenvironment around cells, which promotes proinflammatory responses and inhibits cell division [61]. An inflammatory response is beneficial to eliminate senescent and mutated cells and prevents tumor development and protects bodily functions [7, 62]. In senescence-related research in cancer patients, Srdic-Rajic found that low-dose doxorubicin induced cell senescence and inhibited cancer cell proliferation by promoting ROS production and DNA damage [63]. Further research revealed the appearance of proliferating cells during this process [64]. Later research found that during low-dose chemotherapy, the choice of proliferation or senescence cell fate depended on three different modalities of p21 kinetics. Drug-induced delayed or acute expression of p21 leads to cell senescence, and the intermediate p21 response often results in cell proliferation [65]. Therefore, a p21 “golden zone” was established for the continued proliferation of cells following drug treatment, which provides new guidance for the improvement in clinical chemotherapy strategies and combination medications. Chen et al. found that knockout of the Pten gene resulted in upregulated senescence markers in precancerous tissues but not in deteriorating cancer tissues in a mouse model of prostate cancer. After Pten-deficient cells enter senescence in cell culture, cell senescence is reversed by p53 inactivation [66]. A study using a mouse model with p16^Ink4a^ luciferase labeling to observe cellular senescence and activation in real time revealed that senescent cells accumulated significantly at the site of a transplanted tumor formation in mice; this study represented the first real-time observation of senescent cells in the early stage of cancer *in vivo* [67]. Cellular senescence markers have been employed as early tumor markers in clinical applications.

Senescent cells accumulate in the later stages of tumorigenesis and secrete a large number of inflammatory factors, growth factors, and immunoregulatory factors, which provide an immunosuppressive microenvironment for tumor cells. This microenvironment stimulates tumor cell transformation and promotes tumor cell proliferation, migration, and invasion [68]. Although senescence therapy may be initially beneficial to inhibit the proliferation of tumor cells, it might promote the acceleration of proliferation and malignant transformation of nonsenescent tumor cells from the stimulation and accumulation of cytokines [69]. In the long term, senescent tumor cells might have certain side effects on health [70]. Therefore, combining the treatment of senescence-induced cancer with senolytics may prevent the regrowth of senescent cancer cells [71]. The survival rate of cervical cancer patients was closely related to the level of age-related proteins in the serum; a higher expression of age-related proteins was related to a lower survival rate of patients. Following radiotherapy, the number of senescent cells decreased and the survival rate of cancer patients increased [72]. Together, these studies indicate that both inducing the senescence of cancer cells and the targeted removal of senescent cells could help to fight cancer, and in-depth research into cellular senescence could be significant for cancer prevention and treatment [73].

### 3.2. Atherosclerosis

Vascular senescence induces the development of atherosclerosis. Senescence of vascular smooth muscle and endothelial cells promotes the formation of atherosclerotic plaques [74]. The numbers of mouse bone marrow–derived endothelial progenitor cells decreased with age, and those endothelial cells could not be replaced after peripheral damage. This was attributed to increased inflammation caused by SASP factor stimulation and a reduction in tissue homeostasis and tissue repair mediated by transforming growth factor *β* (TGF-*β*) [75]. Therefore, atherosclerotic lesions appeared in the damaged area. However, damaged blood vessel structures might be repaired better after mice received bone marrow cell transplantation from young healthy donors [76].

An independent study found that senescence foam cells led to increased numbers of macrophages by promoting an inflammatory response. This resulted in an acceleration in the initial course of atherosclerosis and released enzymes and matrix to degrade plaque in the later stages to promote the instability and rupture of fibrous caps [29].

### 3.3. Liver Fibrosis

Senescence of various cell types in the liver has an important role in liver fibrosis [77]. In the fibrotic areas of the liver, the telomeres of hepatocytes are significantly shortened [78]. Senescent liver cells activated surrounding stellate cells to secrete senescence-related active factors, therefore changing the microenvironment in the liver, which aggravated liver fibrosis [79, 80]. However, Krizhanovsky's group studied a mouse model of fibrosis and indicated that the first cells to undergo senescence were activated stellate cells [81]. These cells secrete cytokines to promote natural killer cells to recognize and degrade fibrous tissue and reduce the secretion of extracellular matrix, which effectively limits fibrosis in the liver. However, in p53-deficient murine hepatic fibrosis, continuous activation and proliferation of stellate cells aggravated fibrosis [82]. Therefore, liver fibrosis might be inhibited through the p53 signaling cascade to reverse liver fibrosis [81]. Notably, senescent cells have important physiological and structural functions, such as liver sinusoidal endothelial cells that exhibit important detoxification functions. The researchers used CD31 antibodies to stain the livers of mice with different genotypes and found that the removed senescent sinusoidal endothelial cells were not replaced by new cells (other CD31-positive cells) but promoted tissue fibrosis, which leads to the deterioration of health. The lack of replacement of CD31-positive cells in the liver is due to their low proliferative activity and the decline in the expression of a variety of Vegfs and their receptors due to aging [27].

### 3.4. Neurodegenerative Diseases

In the nervous system, cellular senescence leads to age-related neurodegenerative diseases, including Alzheimer's disease (AD), Parkinson's disease, and amyotrophic lateral sclerosis [83, 84]. Various markers for senescence have been observed in patients with neurodegenerative diseases. Previous studies showed a causal relationship between the accumulation of senescent cells and cognition-related neuronal loss [83]. However, the precise role of senescent cells in the etiology of these neurodegenerative diseases is unknown.

SASP signal activation–mediated neuroinflammation and inflammasome lead to neuron loss [85]. For example, oligodendrocyte precursor cells appear to exhibit a senescence phenotype in AD [86]. Application of senescent cell lysis therapy to AD mouse models led to significantly reduced neuroinflammation and amyloid plaques [87]. In the MAPT^P301S^PS19 model of tau-dependent neurodegenerative disease, the accumulation of p16^INK4A^-positive senescent astrocytes and microglia was observed. The elimination of these cells in INK-ATTAC transgenic mice prevented glial hyperplasia. Elimination of these cells in INK-ATTAC transgenic mice prevented gliosis, deposition of neurofibrillary tangles caused by hyperphosphorylation of soluble and insoluble tau, and degeneration of cortical and hippocampal neurons, thus maintaining cognitive function [88]. Together, these results show the vital role of senescent cells in the initiation and progression of tau-mediated diseases and the therapeutic potential of targeting senescent cells for the treatment of these comorbidities.

### 3.5. Other Age-Related Diseases

The senescence of insulin-secreting *β* cells in the pancreas is related to the progression of type I and type II diabetes and affects the autoimmunity and metabolic functions of the body, respectively [89]. Senescence reduces the proliferation capacity of *β* cells and the secretion of SASP components, thereby aggravating current inflammation and tissue damage [90]. The body loses its ability to keep blood sugar stable under aging, which leads to glucose toxicity [91]. This stress causes the senescence of various types of cells, such as fibroblasts, renal tubular epithelial cells, endothelial cells, and mesenchymal stem cells, which leads to other age-related diseases, such as vascular diseases and kidney diseases [92]. Similar to results in AD, the application of senescent cell lysis therapy in an animal model of diabetes showed promising effects in inhibiting the course of the disease [93].

In idiopathic pulmonary fibrosis, alveolar type II epithelial cells proliferate into new type II epithelial cells or differentiate into type I epithelial cells [94]. However, type II epithelial cells with congenital regeneration defects of short telomeres do not continue to proliferate or differentiate and cannot form normal alveolar tissue. The specific knockout of the type II epithelial cell telomere protection protein TRF2 *in vitro* causes DNA damage response and cell senescence [95]. The DNA damage signal from the alveolar epithelium can recruit macrophages and T cells to the alveolar tissue, and telomere shortening–mediated stem cell senescence upregulates the expression of proinflammatory cytokines and induces inflammation. Senescence increases oxidative stress, which directly leads to DNA damage [96]. Excessive oxidative stress has various adverse effects on cells, such as the activation of redox sensitive signaling pathways and the expression of cytokines and chemokines. Fibroblasts activate and secrete large amounts of collagen fibers, which leads to lung diseases such as idiopathic pulmonary fibrosis [97]. Studies have shown that senescent fibroblasts are selectively killed by dasatinib and quercetin (a senolytic) [98]. Eliminating senescent cells in INK-ATTAC transgenic mice improved lung function and physical health [94].

Osteoarthritis (OA) is a chronic disease characterized by the degradation of articular cartilage, causing pain and physical disability. Studies have found senescent chondrocytes in the cartilage cells of patients with OA and these cells have characteristics of age-related *β*-galactosidase positive staining, shortened telomere length, and mitochondrial degeneration [99]. In a mouse model of OA through anterior cruciate ligament transection, senescent cells are accumulated in the articular cartilage and synovium. Selective removal of these cells reduces the development of OA and relieves pain [100].

## 4. Common Modalities in Cell Senescence and Related Nutritional Interventions

Cellular senescence occurs through long-term culture of primary cells (replication senescence). However, in response to several stress factors (including oxidation, radiation, and toxicity), cellular senescence can be triggered prematurely [101]. In addition to the main senescent pathways, such as p16 and p53-p21, the upregulation of SIRT1, eNOS phosphorylation, SOD, GSH-Px, and E2F-1 and the downregulation of miR-34a, NF-*κ*B, MDA content, and caveolin-1 to delay senescence have been reported in various senescent cell models.

**Figure 4:**
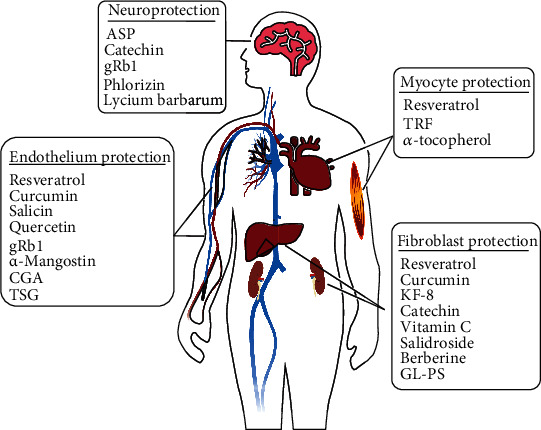
Common research areas for cellular senescence and related nutritional interventions. Many cell models are used to study cellular senescence, and the most widely used cell models are endothelial cells, fibroblasts, muscle cells, and nerve cell models. A variety of plant extracts with effective antiaging properties have been identified. Many herbal extracts exhibit antiaging effects. Natural nutritional interventions for cellular senescence mainly included carbohydrates, polyphenols, peptides, sterol compounds, and vitamins.

Recent research has shown interest in drugs, such as rapamycin and metformin, and their ability to effectively prolong life and treat disease pathology. However, the antiaging ability of these drugs is not unique [102]. From >10,000 screening tests, a variety of plant extracts were identified with potent antiaging properties [103]. Many herbal compounds exhibit anticell senescence effects [104] (Figure 4). The development of new antiaging drugs from natural plants and traditional Chinese medicine has gained global attention [105]. These cell senescence interventions that are extracted from plants include carbohydrates, polyphenols, peptides, sterol compounds, and vitamins (Table 1). Our aim for Table 1 table is to highlight the cells that are suitable for the study of cell senescence, which nutritional interventions can act as positive effectors to interfere with the aging process, which markers can be detected, and whether their results can be compared with those of previous studies.

### 4.1. Endothelial Cells

Vascular endothelial cells (VECs) are the most widely studied cell type. VEC senescence is a common pathological basis for cardiovascular diseases. Under chronic exposure to high glucose (HG) and a high-fat environment, VECs can enter an early stage of senescence. Vascular dysfunction occurs from changes in the levels of vasodilators, contractile factors, antioxidant factors, and coagulation factors [106–110]. After the senescence of HUVECs was cultured *in vitro*, the cells were wider with flat intercellular spaces. In addition, nuclei and nucleoli were enlarged along with reduced levels of nitric oxide (NO) and endothelial nitric oxide synthase (eNOS) activity [111]. NO is a vasodilator factor that promotes blood circulation and helps to control blood pressure. In senescent cells, the production of ROS is significantly increased, which reduces the bioavailability of NO [112]. HG promotes mitochondria to produce excessive ROS, therefore accelerating oxidative damage and cell senescence [113, 114].

Dasatinib combined with quercetin is a recently identified senolytic strategy with pronounced antiaging effects. Dasatinib is a small molecule tyrosine kinase inhibitor. Quercetin is a natural flavonoid and reduces the survival ability of senescent HUVECs to effectively trigger cell death without discernable effects on nonsenescent cells [115]. Curcumin, a natural polyphenol compound, delays endothelial cell senescence induced by hydrogen peroxide through SIRT1 signaling [116]. Recent studies found that a combination of resveratrol, curcumin, and *β*-caryophyllene reduced the levels of SASP factors, such as IL-1*β* and IL-6, in senescent HUVECs [117]. Wang and associates found that Ginseng-Sanqi-Chuanxiong extracts regulated mitosis through AMPK to prevent HG/palmitate-induced endothelial senescence and the production of mitochondrial ROS [118]. New strategic plans are required for the clinical prevention and treatment of cardiovascular-related diseases, especially those related to endothelial cell senescence.

### 4.2. Fibroblasts

The generation of senescent cell models using fibroblasts is a common method to explore the biological characteristics of senescence [119]. High levels of glucose have been used to induce senescence in human diploid fibroblasts [120]. An H_2_O_2_ administration method has been reported to continuously track senescence in primary nonembryonic mouse fibroblasts. After staining with SA-*β*-Gal, the percentage of senescent cells (positively stained for SA-*β*-Gal) in the H_2_O_2_-induced group was 22.23% higher than that in the normal group [121]. Another study used UV to irradiate mouse skin fibroblasts to obtain a skin photo senescence model, and rosiglitazone was found to alleviate senescence in this model [122]. However, other studies used rosiglitazone to induce senescence in bone marrow cells [28]. One possibility for these contrasting findings is that a single drug might have varying effects depending on the cell types.

The senescent fibroblast models are also used for the screening of antisenescence drugs. In a previous study in which bleomycin-induced BJ fibroblasts were used to establish a senescent cell model, the authors screened 113 plant components and obtained several drugs that effectively inhibit SASP formation [123]. Recently, we found that pretreatment with KF-8, a polypeptide extracted from rice bran, delays the H_2_O_2_-induced senescence of 3T3 cells by attenuating NF-*κ*B/p38 signaling and Nrf2 nuclear transport [124]. Another study found that quercetin not only delays the senescence of human primary dermal fibroblasts after UV exposure but also delays the senescence of human primary dermal fibroblasts lacking HES1 (a growth control transcription factor) [ [13]]. Moreover, when quercetin and its derivative quercetin-caprylate was supplemented to senescent fibroblasts, a rejuvenating effect was observed [125]. Although quercetin has good anticellular senescence effects, its poor oral bioavailability, due to poor water solubility, cell membrane permeability, and short biological half-life, limits its clinical application [126]. Other studies have shown the benefit of resveratrol [127, 128] and fisetin [102] from vegetables and fruits in the senescence of fibroblasts.

### 4.3. Muscle Cells

Muscle tissue is mainly composed of highly contractile, columnar muscle cells. The contraction of muscles converts chemical energy into mechanical energy, shortening muscle fibers, which causes various body movements [129]. The senescence of myocardial cells causes a series of physiological and pathological changes in the heart, which lead to the onset of cardiovascular disease, and even mortality in severe cases. Recent evidence suggests that ellagitannins found in pomegranates are converted to Urolitin A in the gut. Urolitin A can slow the senescence of muscle cells by improving mitochondrial function [130]. In another independent study using palmitate to induce muscle cell senescence, resveratrol delayed senescence by altering autophagic flux [131]. The antisenescence ability of resveratrol has been verified in a variety of cell models. Resveratrol has been found to significantly extend lifespan in a variety of model organisms such as yeast, nematodes, fruit flies, fish, mice, and rats [ [132, 133]]. The antisenescence mechanisms of resveratrol mainly involve effects on oxidative stress, calorie restriction, and telomeres. Resveratrol is an antioxidant that ameliorates age-related diseases in mice by reducing ROS production, scavenging free radicals, and stimulating biosynthesis of endogenous antioxidants [134–137]. However, human trials are lacking.

Calorie restriction is the only known nutritional intervention that has the potential to slow down senescence. A recent study in humans has confirmed that cutting calorie intake by 15 percent over two years can slow aging and metabolism, as well as prevent age-related diseases [138]. Resveratrol has been found to have a similar effect to caloric restriction and regulates lifespan through Sir2/SIRT1, AMPK, NF-*κ*B, and other signaling pathways [139].

### 4.4. Nerve Cells

Nerve tissues, composed of signal-transmitting neurons and the supporting glial cells, are basic components of the central and peripheral nervous system. The topic of cell senescence and neuronal regeneration is rapidly evolving in the neuroscience field. The decline of cognitive function and memory is closely associated with the senescence of hippocampal nerve cells and a decrease in the numbers of new neurons during aging [140, 141]. Naturally abundant compounds in plant-based foods have been found to have a wide range of health benefits and may be environmental determinants of brain structure and cognitive function. For example, resveratrol in red grape skin and epigallocatechin gallate (EGCG) in green tea have been shown to influence hippocampal neurogenesis in adults [142, 143]. Recently, quercetin, which is abundant in apple peel, was found to promote hippocampal precursor survival and neuronal differentiation in adult mice. The 3, 5-dihydroxybenzoic acid in apple pulp can significantly increase the proliferation and neurogenesis of neural precursor cells [144]. Curcumin and its analogs was found to reduce oxidative damage of senescent PC12 cells. Curcumin upregulates the level of Nrf2, inhibits ROS production, restores mitochondrial membrane potential, and reduces cell apoptosis [145]. In addition, curcumin increases the level of HO-1 and decreases the expression of Keap1 [146]. In addition to the effects of curcumin in cell models, the antioxidant and antisenescence effects of curcumin have also been verified in animal models such as nematodes and mice [147, 148]. Curcumin not only eliminates ROS and regulates the expression of SOD, catalase, and other related antioxidant enzymes [ [149]] , but it also acts as a calorie restriction mimetic to delay senescence [150]. Although a large number of experiments have shown that curcumin has antisenescence effects, the data on the long-term response to curcumin is still very limited, and clinical verification is still lacking. In addition, curcumin has low bioavailability, and an effective concentration is difficult to achieve, which is also an urgent problem to be solved. We previously reviewed the antisenescence effects of various plant-derived antioxidants on neurons and summarized the mechanism of the effects [151].

## 5. Conclusion and Perspectives

Cellular senescence plays an important role in a variety of pathological processes, including tumorigenesis, atherosclerosis, fibrosis, and the normal aging process. In response to telomere shortening, DNA damage, and external stimuli, senescent cells halt proliferation through various signaling pathways and secrete several factors to attract immune cells for scavenging and tissue regeneration.

Cellular senescence and biological aging are related but are distinct concepts. The study of cellular senescence is moving into a new area to determine the mechanisms of biological (organismal) senescence. Gene cloning technologies and other methods could be employed for the in-depth examination of cellular senescence–related genes to provide a more reliable base for the mechanisms of senescence and senescence-related diseases. Building animal models that mimic human aging diseases also helps further the understanding of the effects of senescent cells on diseases caused by senescence. Various models to explore senescence are currently used in research, including D-galactose induction, thymus removal, and isotope irradiation, which determine the pathological processes of senescence from different perspectives, such as energy metabolism disorders, immune disorders, and DNA damage. The thymus, spleen, serum index, and other indicators are not enough to explain the antisenescence mechanism. The mechanism of cellular senescence is determined by multiple factors, which contribute to the complexity of the modulation of cellular senescence.

Starting from the proven pathways of cellular senescence, cellular senescence modulators that are extracted from natural substances and show clinical relevance to delay cell senescence are being researched. Our group found a polypeptide from rice bran, and its antioxidant and antiaging effects have been proven in cells, nematodes, and mouse models [124]. Future studies should explore the antiaging effect of this peptide on the human body.

Despite the research in drugs targeting senescence, there are still limitations in their application. The first is the low bioavailability of natural compounds. More in-depth pharmacological and pharmacokinetic studies are required to improve the safety, purity, and bioavailability of antiaging drugs and to formulate relevant standards and specifications to ensure the applicability of antiaging drug research [180]. The second limit is that their long-term effects on human health cannot be verified in animal models; the existing animal models and technology cannot evaluate the long-term negative effects caused by clearing or regulating senescent cells. Third, the current research on the mechanism of new antiaging drugs is based on known pathways, and at present, all the mechanisms with antiaging effects remain unknown. Finally, during the research process, it was discovered that certain antiaging effects of certain drugs have sex and age restrictions or are only effective for certain types of cells [181].

Nevertheless, based on the results of current research, antiaging drug research can help identify new antiaging targets or find more effective compounds through modification. Researchers have discovered some senolytic and senomorphic pharmaceutical compounds. Senolytics are mainly effective by eliminating senescent cells, while the function of senomorphics (also called senostatics) is to regulate the characteristics of senescent cells rather than eliminating the cells [5]. The natural interventions mentioned in this article, which have the effect of delaying cell senescence, are expected to become senomorphics through in-depth research. From a long-term perspective, it is feasible to use intervention measures that affect the aging process, such as reducing the load of senescent cells, to delay the onset of age-related onset or decrease the incidence. Therefore, we need to improve the existing models to summarize the various pathological signals of senescence more prominently, to understand the cellular mechanism of senescence, and identify novel interventions that have antisenescence activity from nutrition. In-depth examination of the underlying antisenescence mechanism should help develop new antisenescence interventions such as senolytics and senomorphics for aging and age-related comorbidities.

## Figures and Tables

**Table 1:** Common cellular senescence models and nutritional interventions.

Cell type	Cell line	Induction methods	Inhibitor	Type of inhibitor	Sources (nutritional)	Molecular target(s) (major)	Read-out results	References
Epithelial cells	HUVEC	Ang II	TFs	Polyphenols	Carya cathayensis Sarg	SIRT1↑, miR-34a↓, p53↓, p21↓, PAI-1↓	SA-*β*-GAL positive cells↓, G0/G1 cell cycle↓, S cell cycle↑	[[152]]
	HUVEC	D-galactose	Mulberry extract, C-3-R, C-3-G	Mixture	Mulberry	p21↓, p16↓, NAD+/NADH↑, SIRT1↑	SA-*β*-GAL positive cells↓,ROS↓	[[153]]
	HUVEC	Doxorubicin; replicative	bCUR; Polydatin; BCP	Polyphenol; semiterpenoids	Curcuma aromatica Salisb lemon; grapefruit	SIRT1↑, miR-146a↓, miR-21↓, p16^ink4a^↓, IL-6↓	SA-*β*-GAL positive cells↓,SASP↓	[[117]]
	HUVEC	HG	*α*-Mangostin	Flavonoid derivatives	Mangosteen	IL-6↓, SIRT1↑, AMPK↑, p-AMPK↑, p53↓, p21↓	SA-*β*-GAL positive cells↓,ROS↓	[[154]]
	HUVEC	HG	Aralia elata (Miq.) Seem	Mixture	Aralia elata	p-ERk↑,p-p38↑, cdc2↓,p53↓, cyclinB1↓, SIRT1↑,p-AKT↑, p-AMPK↑, p-eNOS↑	SA-*β*-GAL positive cells↓, G0/G1 cell cycle↓	[[155]]
	HUVEC	H_2_O_2_	TSG	Glycosides	Polygonum multiflorum	SIRT1↑, p21↓, PAI-1↓	SA-*β*-GAL positive cells↓, cell cycle arrest↓	[[156]]
	HUVEC	H_2_O_2_	Curcumin	Polyphenol	Curcuma aromatica Salisb., C. longa L	SIRT1↑, p21↓	SA-*β*-GAL positive cells↓, Cell proliferation↑	[[116]]
	HUVEC	H_2_O_2_	CGA	Phenylpropanoids	Eucommia ulmoides Oliv. Lonicera dasytyla Rehd.	Nrf2↑, HO-1↓, SIRT1↑, PAI-1↓, p21↓, p53↓	SA-*β*-GAL positive cells↓, Cell proliferation↑, DNA damage↓	[[157]]
	HUVEC	H_2_O_2_	Resveratrol	Polyphenols	Grapes, knotweed, peanuts	p-Rb↑, LC3↑, p62↑	SA-*β*-GAL positive cells↓,ROS↓	[[158]]
	HUVEC	Ox-LDL	gRb1	Saponin	Panax ginseng C.A.Mey. P. quinqu efolium L.	SIRT1↑, p62↓, LC3II/LC3I↑, PAI1↓	SA-*β*-GAL positive cells↓, G0/G1 cell cycle↓	[[159]]
	HUVEC	TNF-*α*	Salicin	Organic acid	Willow, Gaultheria, sweet birch	p21↓, PAI-1↓, Acety-p53↓, Nrf2↑	SA-*β*-GAL positive cells↓, cell cycle arrest↓	[[160]]
	HUVEC	Radiation	Quercetin	Flavonoids	Buckwheat, sea buckthorn, hawthorn, onion	PAI-2↓, p21↓, BCL-xL↓, p16↓	SA-*β*-GAL positive cells↓	[[115]]
	HAEC	Ox-LDL	Quercetin	Flavonoids	Buckwheat, sea buckthorn, hawthorn, onion	IGFBP3↓, SLC5A11↑, EIF41B↓	SA-*β*-GAL positive cells↓, ROS↓	[[161]]
	RAEC	HG; PA	GSC extracts	Mixture	Ginseng; San-Qi; Chuan-Xiong	Parkin↑; p21↓, p16↓, p62↓, AMPK↑	MtROS↓, Mitosis↑	[[118]]
	HaCaT	UVA	G6	Polysaccharides	Ascophyllum nodosum	SIRT1↑, pGC1a↑, NRF1↑, NRF2↑, ERRa↑	Respiratory chain complex activities↑, ATP content↑, NAD+/NADH ratio↑	[[162]]
	HaCaT	UVB	SH extracts	Mixture	Salvia haenkei	p21↓, p27↓, IL6↓, IL18↓, SIRT1↑, MMP-2↓	ROS↓	[[163]]
	HaCaT	UVB	SS stem extracts	Mixture	Spatholobus suberectus	p-p38↓, p38↑, ERK1/2↑, p-ERK1/2↓, NF-*κ*B↓	ROS↓, Cell damage↓	[[164]]
Fibroblasts	HDFs	UV	Curcumin	Polyphenols	C. aromatica Salisb., C.longa L	TGF-*β*↑, Smad2/3↑, Bcl-2↑, MMP-1↓, MMP-3↓, caspase-3↓, NF-*κ*B↓, GRP78↓, CHOP↓	ROS↓, activity of antioxidant defense enzymes↑	[[165]]
	HDFs	UVB	Extracts	Mixture	S. aromaticum L.	MMP-1↓, p-c-jun↓, p-c-fos↓ NF-*κ*B↓, I*κ*B-*α*↑, NQO-1↑	ROS↓, Cell viability↑	[[166]]
	HDFs	H_2_O_2_	Vitamin C	Vitamin	Tomatoes, cauliflower, citrus, Grapefruit, apples, grapes	FoxO3a↑, SIRT1↑, p-Rb↓, p53↓, p21↓, p16↓	SA-*β*-GAL positive cells↓, Collagen↑, Elastic fiber↑	[[167]]
	3T3	H_2_O_2_	KF-8	Peptide	Rice bran	Nrf2↑, p65↓	ROS↓	[[124]]
	Fibroblasts	UVB	GL-PS	Polysaccharides	Ganoderma lucidum	MMP-1↓, CICP↑	SA-*β*-GAL positive cells↓, Cell viability↑	[[168]]
	WI-38	CuSO_4_	Resveratrol	Polyphenols	Grapes, knotweed, peanuts	SIRT1↑, p21↓, TGF-*β*↑, ApoJ↓	SA-*β*-GAL positive cells↓, Cell proliferation↑	[[169]]
	2BS	Replicative	Salidroside	Phenyl alcohol	Rhodiola rosea L	PGC-1*α*↑, Nrf1↑, TFAM↑, SIRT1↑, p53↓, p21↓, p16↓, Rb↓	mitochondrial dysfunction↓, ROS↓	[[170]]
	2BS; WI-38	Replicative; H_2_O_2_	Berberine	Alkaloids	Coptis chinensis Franch.	p16↓, CDK4↑, cyclinD1↑, p-RB↑, E2F-1↑, SIRT1↑, p-Chk2↑	SA-*β*-GAL positive cells↓, G0/G1 cell cycle↓, S/G2-M phase↑, ROS↓	[171, 172]
Myocyte	NRCMs	Hypoxia; LPS	Resveratrol	Polyphenols	Grapes, knotweed, peanuts	p53↓, SIRT1↑, p16↓, p19↓, c-Casepase3↓, Bax↓, NLRP3↓	SA-*β*-GAL positive cells↓	[[173]]
Neurocyte	NSCs	D-galactose	ASP	Polysaccharides	Angelica	p53↓, p21↓, TNF*α*↓	SA-*β*-GAL positive cells↓, Cell proliferation↑, ROS↓, activity of antioxidant defense enzymes↑	[[174]]
	NSCs	LiCl	gRb1	Saponin	Panax ginseng C.A.Mey. P. quinqu efolium L.	p-Gsk-3*β*↓, c-myc↓, Lef↓, *β*-catenin↓	SA-*β*-GAL positive cells↓, Cell proliferation↑	[[175]]
	PC12	D-galactose	Phlorizin	Flavonoids	Apples	Nrf2↑, HO-1↑, NQO1↑	SA-*β*-GAL positive cells↓, activity of antioxidant defense enzymes↑	[[176]]
	PC12	H_2_O_2_; AAPH	Ethanol extract of P. ternata tubers	Mixture	Pinellia ternata	p53↑, RPS19BP1↓, HuR↓, SIRT1↑, Bax↓, Bcl-2↑	SA-*β*-GAL positive cells↓, lipofuscin accumulation↓, cell cycle arrest at the G2/M phase↓,oxidative damage↓	[[177]]
	Astrocytes	Replicative LPS/MPP+	Astragaloside IV	Saponin	Astragalus	p16^ink4a^↓, CXCL1↓, IL-6↓, IL-1*β*↓, MMP3↓, Lamin B1↓, p62↓	SA-*β*-GAL positive cells↓, accumulation of senescent astrocytes↓, MtROS↓	[[178]]
Cartilage	Chondrocytes	CCN1	Tanshinone IIA	Ketones	Salvia miltiorrhiza Bge	CCN1↓, p16^ink4a^↓, p21↓, IL-1*β*↓, CXCL1↓, MMP3↓, IL-6↓	SASP↓, ROS↓,	[[179]]

## Data Availability

The data used to support the findings of this study have been deposited in PubMed.
